# Beyond Individual Resilience: A Social–Ecological Perspective on Sustaining the NICU Nursing Workforce

**DOI:** 10.3390/healthcare14111441

**Published:** 2026-05-22

**Authors:** Ji Suk Ryu, So Ra Kang

**Affiliations:** 1 Wonkwang University Hospital, 895 Muwang-ro, Iksan 54538, Republic of Korea; 2 College of Nursing, Wonkwang University, 460 Iksandae-ro, Iksan 54538, Republic of Korea

**Keywords:** neonatal intensive care unit, NICU nurses, job satisfaction, resilience, communication competence, nursing work environment, social–ecological model

## Abstract

**Background/Objectives:** Sustaining a stable and competent workforce in neonatal intensive care units (NICUs) is critical for ensuring high-quality care for vulnerable neonates. However, workforce-related challenges such as job dissatisfaction and turnover remain significant concerns in high-acuity settings. Guided by the ecological model, this study aimed to examine resilience, communication competence, and the nursing work environment as multilevel factors associated with nursing workforce sustainability, with job satisfaction serving as a proxy indicator related to workforce retention and sustainability. **Methods:** A descriptive cross-sectional survey was conducted among 145 NICU nurses from three tertiary and three general hospitals in South Korea. Data were analyzed using descriptive statistics, independent *t*-tests, one-way ANOVA, Pearson’s correlation coefficients, and multiple regression analyses using SPSS version 29.0. **Results:** Job satisfaction was positively associated with resilience (*r* = 0.67, *p* < 0.001), communication competence (*r* = 0.52, *p* < 0.001), and the nursing work environment (*r* = 0.57, *p* < 0.001). Multiple regression analysis indicated that resilience (*β* = 0.43, *p* < 0.001), nursing work environment (*β* = 0.30, *p* < 0.001), communication competence (*β* = 0.15, *p* = 0.040), and employment in a tertiary hospital (*β* = 0.12, *p* = 0.038) were significant factors associated with job satisfaction, explaining 55.1% of the variance (adjusted R^2^) in job satisfaction (F = 30.42, *p* < 0.001). **Conclusions:** Job satisfaction, used as a proximal indicator related to workforce sustainability, was associated with multilevel factors across intrapersonal, interpersonal, and organizational domains. Although resilience showed the strongest association, communication competence and the nursing work environment also showed meaningful associations with job satisfaction. These findings highlight the need for integrated, multilevel strategies to support nursing workforce sustainability and sustained nursing practice in NICU settings.

## 1. Introduction

In South Korea, the number of births has steadily declined in recent years. In 2023, the total number of births was 230,028, representing a decrease of 19,200 compared with the previous year, and the total fertility rate declined to 0.72, which was 0.06 lower than the previous year [1]. Despite this decline, the proportion of high-risk newborns has increased, largely due to rising maternal age and the growing use of assisted reproductive technologies [2,3]. Consequently, both preterm births (gestational age < 37 weeks) and low-birth-weight infants (birth weight < 2.5 kg) have increased substantially over the past decade, with rates in South Korea reaching 9.9% and 7.7%, respectively [1,4]. Preterm and low-birth-weight infants often have unstable physiological functions and immature central nervous systems, making them highly vulnerable to extrauterine adaptation immediately after birth and therefore requiring intensive monitoring, treatment, and individualized nursing care in the NICU [5].

Advances in medical technology have improved the survival rates of premature and high-risk neonates, thereby increasing the importance of NICUs as critical care settings for continuous physiological monitoring and timely clinical intervention [4]. NICU nurses provide individualized, condition-specific care tailored to the complex health needs of neonates [6]. They manage neonates with complex medical and developmental conditions requiring continuous monitoring and specialized care [5]. Additionally, neonates are highly vulnerable to infection because of their immature immune systems and increased permeability of the skin and mucous membranes, underscoring the necessity of strict infection prevention practices in NICU settings [7].

NICU nurses often work in emotionally demanding environments. Despite providing advanced care using modern medical technologies, they frequently encounter situations in which the prognosis of neonates is poor or survival is uncertain, reflecting the inherent uncertainty in neonatal intensive care settings [8,9,10]. Because NICU nurses care for newborns at the beginning of life, they may become emotionally involved in the care process and support the development of mother–infant attachment [11]. When adverse outcomes occur, nurses may experience moral distress and associated emotional burdens, such as guilt and helplessness, particularly when neonatal death is perceived as a failure of care [12]. In this context, NICU nurses not only play a key role in delivering direct care but also in coordinating communication among healthcare professionals and families [13]. Therefore, understanding factors associated with the job satisfaction of NICU nurses is important for supporting a stable and sustainable NICU nursing workforce and maintaining high-quality neonatal care.

The characteristics of NICU nursing require continuous observation and care for neonates who cannot verbally communicate their needs, which increases both physical fatigue and psychological stress among nurses [14]. Resilience is commonly defined as the ability to adapt to adversity, recover from stressful situations, and achieve positive outcomes despite challenges [15]. Nurses with higher resilience tend to cope with stress through proactive and optimistic attitudes, which may contribute to more effective coping and improved work performance [16]. In addition, higher resilience has been associated with increased self-esteem, stronger work engagement, and greater job satisfaction among nurses in previous studies [17,18]. Importantly, resilience can be enhanced through educational and training interventions [19], suggesting its potential relevance to job satisfaction and workforce stability in NICU settings.

Another important factor in NICU nursing practice is communication competence. In NICU settings, nurses play a central role in providing comprehensive and compassionate care for physiologically vulnerable neonates and their families [20,21]. Effective care for high-risk neonates requires multidisciplinary collaboration, and nurses continuously communicate with physicians, fellow nurses, and healthcare professionals from various departments [22,23]. Communication competence refers to the ability to express one’s thoughts clearly and effectively through appropriate interpersonal interactions [24,25]. Nurses with higher communication competence are more likely to establish supportive relationships with colleagues and receive greater professional support [26]. Furthermore, effective communication competence can enhance work efficiency and improve nursing performance, making it an essential component of high-quality nursing care and collaborative NICU practice [27,28].

In addition to individual and interpersonal factors, organizational factors such as the nursing work environment play a crucial role in nurses’ professional experiences and outcomes [29]. NICU nurses perform both direct and indirect nursing activities while managing complex and sophisticated medical equipment as part of their routine clinical workload [30]. The nursing work environment is defined as the organizational characteristics that support nurses in providing professional nursing care and enable effective clinical practice [31]. A positive nursing work environment facilitates clinical decision-making and professional judgment among nurses, thereby enhancing care quality [32]. Previous studies have shown that favorable nursing work environments improve patient care quality and increase nurses’ job satisfaction [33]. In NICU settings, supportive nursing work environments have also been associated with higher levels of family-centered care and stronger intentions to remain in the organization [34,35], highlighting their critical role in sustaining a stable and competent nursing workforce.

Job satisfaction is considered an important indicator related to workforce sustainability because it is closely associated with nurses’ retention and organizational commitment [36,37]. Higher job satisfaction has also been consistently associated with lower turnover intention and greater retention among nurses, which may contribute to long-term workforce stability [36,37,38]. Higher job satisfaction among nurses has been associated with improved nursing performance and higher-quality patient care [38,39]. In NICU settings, the quality of care is particularly closely linked to nurses’ clinical experience and competencies, as they must respond to diverse and complex neonatal conditions [40,41,42]. However, staffing challenges remain a significant issue. Although developing expert-level clinical competence typically requires more than five years of experience [43,44], a substantial proportion of NICU nurses in Korea are relatively inexperienced, with approximately 42% having less than three years of clinical experience [45]. Furthermore, the resignation rate among nurses with less than three years of experience has been reported to reach 59.1% [46]. Taken together, these findings highlight the urgent need to enhance job satisfaction as a key strategy for sustaining a stable and competent NICU nursing workforce. In the present study, workforce sustainability was conceptualized as the capacity to maintain a stable and retained nursing workforce over time, particularly by supporting nurses’ continued engagement and retention in demanding clinical settings. Because actual retention outcomes require longitudinal observation, job satisfaction was conceptualized as a proximal indicator associated with workforce sustainability [35,36,37,38].

Previous studies have identified various factors related to nurses’ job satisfaction, including resilience, communication competence, and the nursing work environment, as well as emotional intelligence, positive psychological capital, self-efficacy, and professional self-concept [17,18,26,35,47,48,49]. Despite this growing body of evidence, studies focusing specifically on NICU nurses remain relatively limited, highlighting the need for further research in this area [40]. Furthermore, previous studies have often focused on individual or limited organizational factors, which may not fully capture the complex multilevel interactions associated with workforce sustainability in high-acuity settings such as the NICU.

The ecological model provides a comprehensive framework for understanding complex phenomena in healthcare by conceptualizing outcomes as emerging from the interplay between individuals and their surrounding environments. According to McLeroy et al. [50], factors associated with behaviors and outcomes can be categorized into multiple levels, including intrapersonal, interpersonal, and organizational factors. This multilevel perspective is particularly relevant in healthcare settings, where nurses’ experiences are shaped by personal capacities, social relationships, and organizational contexts [26,33,38,50]. Although the social–ecological model encompasses broader community- and policy-level influences, the present study selectively focused on intrapersonal, interpersonal, and organizational factors that were considered most directly relevant and measurable within the NICU workplace context [50]. This framework highlights that outcomes such as job satisfaction are shaped not by single factors but by the combined influence of factors across multiple levels [38]. Applying an ecological perspective therefore enables a more integrated understanding of factors associated with workforce sustainability in NICU settings, where high-acuity care requires the integration of individual resilience, communication competence, and supportive work environments [26,33].

Therefore, this study aimed to examine multilevel factors associated with job satisfaction as a proximal indicator related to workforce sustainability among NICU nurses using an ecological perspective. Resilience, communication competence, and the nursing work environment were examined as key multilevel determinants across intrapersonal, interpersonal, and organizational levels. By examining how these multilevel factors are collectively associated with job satisfaction as a key indicator of workforce sustainability, this study extends the application of the ecological model in healthcare settings and provides a more integrated understanding of workforce sustainability in high-acuity environments. Ultimately, the findings may inform integrated strategies to support workforce sustainability and high-quality care in NICU settings.

## 2. Materials and Methods

### 2.1. Study Design

This study employed a descriptive cross-sectional survey design to examine the levels of resilience, communication competence, nursing work environment, and job satisfaction among NICU nurses and to examine the associations of multiple factors with job satisfaction as a proximal indicator of workforce sustainability within an ecological framework. Community- and policy-level determinants were not included because the present study focused specifically on factors that were considered most directly relevant and measurable within the NICU workplace context.

### 2.2. Participants

The participants were nurses working in NICUs in tertiary and general hospitals designated as neonatal intensive care centers by the Ministry of Health and Welfare in Korea. The hospitals were located in the Seoul, Gyeonggi, Chungcheong, and Jeolla regions of South Korea. Nurses who had at least six months of NICU work experience were included, as this period is generally required for adaptation to the clinical environment [51,52]. Eligible participants were nurses who directly provided care to preterm or high-risk neonates and voluntarily agreed to participate after receiving information about the study.

The required sample size was calculated using G*Power version 3.1.9.7. Based on a significance level of 0.05, an effect size of 0.15, a statistical power of 0.80, and 13 predictors for multiple regression analysis, the minimum sample size was determined to be 131. Considering a potential dropout rate of 10%, a total of 144 participants were targeted. Of the 147 questionnaires collected, two incomplete responses were excluded, resulting in 145 questionnaires being included in the final analysis.

### 2.3. Measures

Data were collected using a structured self-administered questionnaire consisting of five sections: general characteristics, resilience, communication competence, nursing work environment, and job satisfaction. Permission to use the measurement tools was obtained from the original developers prior to data collection. The questionnaire consisted of a total of 112 items, including 10 items on general characteristics, 25 items on resilience, 15 items on communication competence, 29 items on nursing work environment, and 33 items on job satisfaction. Content validity of the modified instruments was evaluated by an expert panel consisting of seven experts, including two professors of pediatric nursing, one doctoral researcher in pediatric nursing, and four experienced NICU nurses (three with over 10 years of clinical experience and one with over eight years of experience). Minor wording modifications were made to several items to improve clarity and contextual relevance for NICU nurses while preserving the original conceptual meaning of each instrument. These modifications primarily involved adapting expressions to reflect the NICU clinical environment and nursing practice context. According to the criteria suggested by Polit et al. [53], all instruments met the recommended thresholds for content validity (I-CVI ≥ 0.78 and S-CVI/Ave ≥ 0.90). Because the instruments were adapted through minor contextual wording revisions rather than substantive structural modifications, the original construct structure of the instruments was retained.

#### 2.3.1. Resilience

Resilience was measured using the Korean version of the Connor–Davidson Resilience Scale (K-CD-RISC) [54], originally developed by Connor and Davidson [55]. The scale consists of 25 items across five subdomains: hardiness (9 items), persistence (8 items), optimism (4 items), support (2 items), and spirituality (2 items). Each item is rated on a 5-point Likert scale (0–4) ranging from 0 (“not true at all”) to 4 (“true nearly all the time”), with higher scores indicating greater resilience. The original scale demonstrated good reliability (Cronbach’s α = 0.89), and the reliability in this study was Cronbach’s α = 0.92.

#### 2.3.2. Communication Competence

Communication competence was measured using the Global Interpersonal Communication Competence Scale (GICC) developed by Hur [56] and modified by Lee and Kim [57] for use among nurses. The scale consists of 15 items assessing various dimensions of communication competence, including self-disclosure, empathy, social relaxation, assertiveness, concentration, interaction management, expressiveness, supportiveness, immediacy, efficiency, social appropriateness, conversational coherence, goal detection, responsiveness, and noise control. Each item is rated on a 5-point Likert scale, with higher scores indicating higher communication competence. Two items were reverse scored. Previous studies reported acceptable reliability (Cronbach’s α = 0.72–0.83), and the reliability in this study was Cronbach’s α = 0.89.

#### 2.3.3. Nursing Work Environment

The nursing work environment was measured using the Korean version of the Practice Environment Scale of the Nursing Work Index (PES-NWI), originally developed by Lake [58] and translated by Cho et al. [59]. The scale consists of 29 items across five domains: nurse participation in hospital affairs (9 items), nursing foundations for quality of care (9 items), nurse manager ability, leadership, and support (4 items), staffing and resource adequacy (4 items), and collegial nurse–physician relations (3 items). Several items were slightly modified to reflect the NICU context. Each item is rated on a 4-point Likert scale ranging from 1 (“strongly disagree”) to 4 (“strongly agree”), with higher scores indicating a more positive perception of the nursing work environment. The original and Korean versions demonstrated good reliability (Cronbach’s α = 0.82 and 0.93, respectively), and Cronbach’s α in this study was 0.93.

#### 2.3.4. Job Satisfaction

Job satisfaction was measured using the Job Satisfaction Scale for Clinical Nurses (JSS-CN) developed by Lee et al. [60]. The scale consists of 33 items across six subdomains, including organizational recognition and professional achievement, human maturity through nursing professionalism, interpersonal relationships based on respect and recognition, fulfillment of professional responsibility, demonstration of professional competence, and job stability and reward. Each item is rated on a 5-point Likert scale ranging from 1 (“strongly disagree”) to 5 (“strongly agree”), with higher scores indicating greater job satisfaction. Content validity of the modified items was evaluated by an expert panel, and all items met acceptable validity criteria. The original instrument demonstrated excellent reliability, with a Cronbach’s α of 0.95 at the time of development by Lee et al. [60]. In the present study, the reliability of the scale was Cronbach’s α = 0.96.

### 2.4. Data Analysis

Data were analyzed using SPSS version 29.0 (IBM Corp., Armonk, NY, USA). Descriptive statistics, including frequencies, percentages, means, and standard deviations, were used to summarize participants’ general characteristics and the levels of resilience, communication competence, nursing work environment, and job satisfaction. Differences in study variables according to participants’ general characteristics were examined using independent *t*-tests or one-way analysis of variance (ANOVA), as appropriate, and Scheffé’s test was performed for post hoc comparisons. Pearson’s correlation coefficients were calculated to examine the relationships among resilience, communication competence, nursing work environment, and job satisfaction. Multiple regression analysis was conducted to identify factors associated with job satisfaction among NICU nurses. Statistical significance was set at *p* < 0.05.

### 2.5. Ethical Considerations

Ethical approval for this study was obtained from the Institutional Review Board of W University (WKIRB-202508-SB-059), and data were collected from 28 August to 2 October 2025 after receiving IRB approval. All participants were informed about the study purpose and procedures before participation and provided written consent. Data were collected anonymously, and participants were assured of their right to withdraw at any time without any disadvantage.

## 3. Results

### 3.1. General Characteristics of the Participants

The general characteristics of the participants are presented in Table 1. A total of 145 NICU nurses were included in the analysis. The mean age of the participants was 30.61 ± 5.68 years, and 76 nurses (52.4%) were aged <30 years. Most participants were unmarried (67.6%) and held a bachelor’s degree (79.3%). Regarding the type of institution, 89 nurses (61.4%) worked in tertiary hospitals and 56 (38.6%) in general hospitals. Most participants were staff nurses (90.3%). The mean total clinical experience was 6.45 ± 5.88 years, and the largest proportion of participants (30.3%) had less than 3 years of total clinical experience. The mean NICU work experience was 3.74 ± 3.41 years, with the largest group (39.3%) having 1–<3 years of NICU experience. Most participants worked rotating shifts (98.6%). Regarding the number of assigned newborns per day, 45.6% cared for four newborns, followed by ≤3 newborns (37.2%) and ≥5 newborns (17.2%). More than half of the participants (52.4%) reported their health status as average, followed by healthy (36.6%) and unhealthy (11.0%).

### 3.2. Levels of Resilience, Communication Competence, Nursing Work Environment, and Job Satisfaction

The mean scores were 2.42 ± 0.47 for resilience (out of 4), 3.82 ± 0.47 for communication competence (out of 5), 2.85 ± 0.41 for the nursing work environment (out of 4), and 3.58 ± 0.63 for job satisfaction (out of 5) (Table 2).

### 3.3. Differences in Study Variables According to General Characteristics

Resilience differed significantly according to subjective health status (F = 13.82, *p* < 0.001) (Table 3). Post hoc analysis indicated that nurses who perceived themselves as healthy had higher resilience scores than those who perceived themselves as average or unhealthy. Communication competence differed significantly according to age (t = 2.45, *p* = 0.016), marital status (t = 2.34, *p* = 0.021), and subjective health status (F = 6.83, *p* = 0.001). Nurses younger than 30 years and unmarried nurses reported significantly higher communication competence. In addition, those who perceived themselves as healthy or average showed significantly higher communication competence than those who perceived themselves as unhealthy. The nursing work environment differed significantly according to age (t = 2.55, *p* = 0.012), type of institution (t = 3.30, *p* = 0.001), total clinical experience (F = 3.74, *p* = 0.013), and subjective health status (F = 5.49, *p* = 0.005). Younger nurses, those working in tertiary hospitals, and those with better perceived health status reported more positive perceptions of the nursing work environment. Job satisfaction differed significantly according to type of institution (t = 3.17, *p* = 0.002) and subjective health status (F = 7.96, *p* = 0.001). Nurses working in tertiary hospitals reported higher job satisfaction than those working in general hospitals. In addition, nurses who perceived themselves as healthy or average showed significantly higher job satisfaction than those who perceived themselves as unhealthy.

### 3.4. Correlations Among Resilience, Communication Competence, Nursing Work Environment, and Job Satisfaction

The correlations among resilience, communication competence, nursing work environment, and job satisfaction are presented in Table 4. Job satisfaction was positively correlated with resilience (*r* = 0.67, *p* < 0.001), communication competence (*r* = 0.52, *p* < 0.001), and the nursing work environment (*r* = 0.57, *p* < 0.001). Communication competence was also positively correlated with resilience (*r* = 0.61, *p* < 0.001). In addition, the nursing work environment was positively correlated with resilience (*r* = 0.44, *p* < 0.001) and communication competence (*r* = 0.35, *p* < 0.001).

### 3.5. Factors Associated with Job Satisfaction

Multiple regression analysis was conducted to identify factors associated with job satisfaction among NICU nurses (Table 5). Variables entered into the model included type of institution and subjective health status, which showed significant differences in job satisfaction in the univariate analyses, as well as resilience, communication competence, and nursing work environment, which were significantly correlated with job satisfaction. Type of institution and subjective health status were entered as dummy variables, with general hospitals and unhealthy subjective health status serving as the reference categories, respectively. The assumptions for multiple regression were satisfied. The Durbin–Watson statistic was 1.928, indicating no serious autocorrelation. Multicollinearity was not a concern, as the variance inflation factor ranged from 1.090 to 3.296 and tolerance values ranged from 0.303 to 0.918. The overall regression model was statistically significant (F = 30.42, *p* < 0.001), and the explanatory variables accounted for 55.1% of the variance in job satisfaction. Resilience showed the strongest association with job satisfaction (*β* = 0.43, *p* < 0.001), followed by the nursing work environment (*β* = 0.30, *p* < 0.001), communication competence (*β* = 0.15, *p* = 0.040), and working in a tertiary hospital (*β* = 0.12, *p* = 0.038). Although subjective health status showed differences in job satisfaction in the univariate analysis, it was not independently associated with job satisfaction.

Overall, job satisfaction among NICU nurses was significantly associated with intrapersonal, interpersonal, and organizational factors, with resilience identified as the factor most strongly associated with job satisfaction within the ecological model framework.

## 4. Discussion

This study examined workforce sustainability among NICU nurses through an ecological lens, conceptualizing job satisfaction as a proximal indicator of workforce retention in high-acuity settings [36,37,38]. The findings suggest that job satisfaction is associated with the combined influence of intrapersonal, interpersonal, and organizational factors, rather than being explained solely by any single determinant, consistent with the core assumptions of the ecological model [38,50]. The relatively substantial explanatory power of the model further suggests that intrapersonal, interpersonal, and organizational factors collectively provide an important framework for understanding job satisfaction among NICU nurses in high-acuity clinical settings. In particular, resilience showed the strongest association, followed by the nursing work environment and communication competence, highlighting the relative importance of individual adaptive capacity alongside contextual and relational conditions. These findings are in line with previous studies reporting that resilience, supportive work environments, and communication competence are positively associated with job satisfaction and nurse retention across clinical settings [17,26,33,35].

This study identified key factors associated with nurses’ job satisfaction, highlighting its importance as an outcome reflecting workplace experiences. In interpreting these findings, it is important to clarify the conceptual relevance of job satisfaction in relation to workforce sustainability. Although job satisfaction is not a direct measure of workforce sustainability, it is widely recognized as a proximal indicator associated with nurse retention [35,36,37,38,39]. Given that actual turnover and retention outcomes typically require longitudinal observation, the use of job satisfaction provides a practical and theoretically justified proxy for workforce sustainability, particularly in cross-sectional research designs. Accordingly, the present findings may have implications for workforce retention in NICU settings. Nevertheless, this proxy-based interpretation should be approached with caution, as job satisfaction reflects a proximal rather than definitive measure of long-term workforce sustainability.

Differences in job satisfaction according to general characteristics provide additional insight into contextual conditions that may be associated with the sustainability of the NICU nursing workforce. In this study, job satisfaction was significantly higher among nurses working in tertiary hospitals than among those working in general hospitals. This finding is consistent with previous studies indicating that more favorable organizational conditions, including adequate staffing, resource availability, and institutional support, are associated with higher job satisfaction and improved nurse outcomes [33,35]. These differences may reflect the greater availability of organizational and professional support in tertiary hospitals. Given the high acuity and complexity of neonatal care, such institutional support may be related to nurses performing their roles more effectively and experiencing greater professional fulfillment, which may be associated with higher job satisfaction. In high-acuity NICU settings, these structural advantages may help reduce workload-related strain and support clinical practice [29,33].

In addition, subjective health status emerged as a consistent factor associated not only with job satisfaction but also with resilience, communication competence, and perceptions of the nursing work environment. Nurses who perceived themselves as healthy reported more favorable outcomes across these variables, suggesting that perceived health may reflect an important condition related to personal coping capacity and professional engagement. This finding is consistent with previous studies indicating that better perceived health is associated with lower burnout, higher job satisfaction, and improved work-related outcomes among nurses [29,33]. In high-intensity environments such as NICUs, where physical fatigue and emotional strain are substantial, maintaining personal health may be associated with more positive work experiences.

Although differences in communication competence and work environment were observed according to age and marital status, these factors did not directly translate into differences in job satisfaction in the multivariate model. This finding is consistent with previous studies suggesting that demographic characteristics are often indirectly associated with job satisfaction through work-related and psychological factors rather than showing direct associations [17,33]. This suggests that while demographic characteristics may shape certain competencies or perceptions, their influence on job satisfaction—and by extension, workforce sustainability—may be indirect and mediated through more proximal factors such as resilience and organizational conditions. Overall, these findings highlight that workforce sustainability in NICU may be shaped by a combination of institutional context and nurses’ perceived well-being rather than by individual demographic characteristics alone.

Resilience, representing the intrapersonal level within the ecological framework, emerged as the strongest factor associated with job satisfaction, underscoring its central role in sustaining the NICU nursing workforce. This finding may reflect the uniquely high emotional and clinical demands of NICU settings, where nurses are continuously exposed to critically ill neonates, uncertain prognoses, and emotionally challenging care situations requiring ongoing adaptation [8,9,10,11,12,13,14]. In such environments, resilience may function as a key adaptive resource associated with nurses’ ability to maintain emotional stability, professional engagement, and positive perceptions of their work despite ongoing stress [15,16,17,18].

Resilience may also be associated with nurses’ ability to regulate emotional responses, recover from distress, and maintain professional engagement despite ongoing emotional demands. The present findings are consistent with previous international and Korean studies reporting positive associations between resilience and job satisfaction, reduced burnout, enhanced coping strategies, and greater work engagement among nurses in high-stress clinical settings [17,18,29]. Notably, the relatively strong association observed for resilience in this study may reflect the unique characteristics of NICU practice, where emotional demands are particularly intense and continuous. In this regard, resilience may function as an important factor associated with sustained professional engagement over time. Although organizational factors were also significant, individual resilience may function as a foundational adaptive resource enabling nurses to maintain psychological stability within demanding clinical environments.

Importantly, the prominence of resilience in this study suggests that efforts to enhance workforce sustainability may benefit from extending beyond structural improvements to include strategies aimed at strengthening nurses’ adaptive capacities. This perspective is supported by prior research demonstrating that resilience-building interventions are associated with improved coping, reduced burnout, and enhanced job satisfaction among nurses [17,29]. While organizational support remains critical, individual-level interventions—such as resilience training, reflective practice, and psychological support programs—may be associated with nurses’ ability to better navigate the complex emotional landscape of NICU care. However, resilience should not be viewed as a substitute for adequate organizational support; rather, it should be understood as one component of broader efforts to support nurses in demanding clinical environments [38,50].

Communication competence was identified as a significant factor associated with job satisfaction, highlighting the importance of relational dynamics in NICU nursing practice. Communication competence is inherently relational and has been associated with collaborative practice and improved work outcomes in nursing settings [26,33]. NICU nurses operate in settings characterized by high clinical uncertainty and emotional intensity, requiring frequent coordination with multidisciplinary teams and sensitive communication with parents of critically ill neonates. In such contexts, communication competence extends beyond information transfer to include emotional attunement, conflict management, and the co-construction of shared understanding, all of which are fundamentally relational processes.

The positive association between communication competence and job satisfaction observed in this study is consistent with prior research demonstrating that communication competence is linked to improved teamwork, reduced interpersonal conflict, and enhanced professional relationships among nurses [26,33]. In both international and Korean studies, communication competence has been associated with greater collaboration and lower work-related stress, suggesting that its benefits are largely mediated through improved relational functioning [26,35]. In the NICU context, where interactions are often emotionally charged and clinically complex, these relational benefits may be particularly salient in shaping nurses’ work experiences.

Notably, communication competence may function as a stabilizing interpersonal factor associated with trust, predictability, and mutual support within the clinical team. This interpretation is supported by prior studies indicating that communication competence is associated with team cohesion, reduced conflict, and improved work environments in nursing settings [26,33]. By facilitating clearer exchanges and more constructive interactions, it may be associated with reduced relational strain and a more cohesive work environment, thereby being linked to higher job satisfaction. These findings suggest that efforts to support workforce sustainability should not conceptualize communication competence merely as an individual trait to be strengthened, but rather as a relational capacity that can be cultivated through team-based and interprofessional interventions. This interpretation aligns with international evidence supporting communication-focused and team-based interventions in nursing settings [26,35]. Overall, these findings highlight the importance of communication competence in collaborative NICU practice.

The nursing work environment was identified as a significant factor associated with job satisfaction, highlighting the critical role of structural and contextual conditions in NICU nursing practice. A favorable work environment involves conditions that support professional practice, including adequate staffing, supportive leadership, opportunities for participation in decision-making, and collaborative relationships, all of which are associated with a more positive evaluation of one’s work [33]. Previous international and Korean studies have similarly shown that supportive work environments, adequate staffing, and organizational support are associated with job satisfaction, nurse retention, and workforce stability in intensive care settings [33,34,35]. In high-acuity settings such as NICUs, these organizational factors may be even more closely associated with nurses’ work experiences, as nurses are required to manage complex clinical situations while ensuring continuous monitoring and rapid responses to neonatal deterioration. In this context, a supportive work environment may reduce workload-related strain, enhance clinical autonomy, and facilitate effective teamwork, thereby supporting more stable and effective nursing practice.

In NICU settings, challenges related to staffing levels and resource availability are commonly reported, suggesting that perceived insufficiency of personnel and resources may remain a critical issue. This is consistent with previous research indicating that inadequate staffing is strongly associated with work overload, burnout, and turnover intention among nurses [29,33]. Even when other aspects of the work environment are relatively favorable, limitations in staffing and resources may undermine nurses’ ability to provide high-quality care and may be associated with lower job satisfaction. These findings highlight staffing- and resource-related conditions as important organizational priorities in high-acuity NICU settings.

Taken together, the findings of this study provide empirical support for the ecological model by demonstrating that job satisfaction—conceptualized as a key indicator of workforce sustainability—is associated with the combined influence of intrapersonal, interpersonal, and organizational factors [38,50]. Rather than operating in isolation, resilience, communication competence, and the nursing work environment each showed unique associations with job satisfaction, suggesting that workforce sustainability in NICU settings may emerge from the combined contribution of multilevel resources. This integrative perspective extends prior research that has often focused on single factors by highlighting the need to consider the dynamic interplay between individual capacity, relational processes, and organizational conditions. These findings underscore the importance of adopting a multilevel ecological approach to support job satisfaction and sustain the NICU nursing workforce and may have broader relevance to international efforts aimed at workforce sustainability in high-acuity healthcare settings.

The differential contributions of these factors highlight the importance of considering individual, interpersonal, and organizational conditions together when interpreting job satisfaction among NICU nurses. While resilience showed the strongest association with job satisfaction, its role should be understood within a broader context in which interpersonal and organizational supports are associated with individual adaptive capacity. Similarly, communication competence is associated with collaborative functioning and relational stability, but its effectiveness may depend on the presence of a work environment that fosters open communication and mutual support. In this regard, the nursing work environment may serve as a foundational context associated with the expression and impact of both individual and interpersonal resources.

These results indicate that interventions aimed at improving job satisfaction and sustaining the NICU nursing workforce should move beyond single-level approaches. Programs that focus exclusively on strengthening individual resilience may be insufficient if organizational constraints remain unaddressed. Likewise, improvements in the work environment may not necessarily be associated with better outcomes without attention to interpersonal and individual competencies. These findings support the need for interventions addressing both organizational conditions and interpersonal support in NICU settings.

The present findings may inform efforts to improve nurses’ work experiences and retention in NICU settings. Accordingly, efforts to strengthen workforce sustainability should prioritize interventions that enhance job satisfaction by addressing factors across ecological levels. This perspective highlights job satisfaction as an important leverage point for translating multilevel interventions into sustainable workforce outcomes in high-acuity settings.

From a practical perspective, the findings suggest that multilevel interventions are needed in NICU settings. At the intrapersonal level, resilience-building interventions, such as reflective practice programs, stress management training, and psychological support, may help nurses better adapt to the emotional demands of NICU care. At the interpersonal level, communication-focused interventions, including simulation-based training and interprofessional education, may be associated with enhanced collaborative practice and reduced relational strain. At the organizational level, improvements in the nursing work environment—such as staffing levels, resource availability, leadership support, and opportunities for nurse participation—should be considered a fundamental priority. Importantly, the observed differences by institution type indicate that targeted organizational support may be particularly needed in general hospital settings to reduce disparities in job satisfaction.

Several limitations should be considered when interpreting the findings. First, job satisfaction was used as a proxy indicator of workforce sustainability. Although it is theoretically grounded and widely recognized as a factor associated with turnover intention and retention, it represents a proximal rather than definitive measure of long-term workforce sustainability and should therefore be interpreted with caution. Second, although this study was guided by an ecological framework, not all relevant levels and contextual factors within the ecological model were fully captured, which may limit the comprehensiveness of the analysis. Because broader healthcare system and policy-related determinants were not fully included, the findings should be interpreted as reflecting a partial application of the ecological framework. Third, the cross-sectional design limits causal inferences regarding the relationships among variables. Fourth, this study used convenience sampling and included NICU nurses from selected regions of South Korea; thus, caution is needed when generalizing the findings. Hospitals with different staffing systems, organizational cultures, resource availability, and regional characteristics may demonstrate different patterns of association. Further studies involving more diverse hospital settings and broader geographic regions are warranted. Fifth, all variables were measured using self-reported questionnaires, which may be subject to response bias. Although content validity was established through expert review, further psychometric validation of the context-adapted instruments will be important in future studies. Despite these limitations, this study provides meaningful insights by applying an ecological framework to a relatively understudied population of NICU nurses and by examining multilevel factors simultaneously.

Future longitudinal studies are needed to examine how multilevel factors relate to job satisfaction, retention, and workforce outcomes over time. In addition, further studies are needed to explore potential mediating and moderating mechanisms among intrapersonal, interpersonal, and organizational variables. Expanding outcome variables beyond job satisfaction to include turnover intention, retention, burnout, and professional quality of life would provide a more comprehensive understanding of workforce sustainability. Finally, given the specialized nature of NICU practice, the development and validation of context-specific measurement tools will be important for advancing research and improving the precision of findings in this field.

## 5. Conclusions

This study demonstrated that job satisfaction, as a key indicator of workforce sustainability, is associated with multilevel factors, including resilience, communication competence, and the nursing work environment. Among these, resilience showed the strongest association, highlighting the importance of intrapersonal capacity in sustaining NICU nursing practice. These results indicate that workforce sustainability may not be adequately supported through single-level interventions. Instead, integrated strategies that enhance individual resilience, strengthen relational communication, and improve organizational work environments may support nurses’ sustained engagement and retention in high-acuity settings. An ecological perspective may provide a useful framework for understanding and supporting workforce sustainability in NICU settings.

## Figures and Tables

**Table 1:** General characteristics of the participants (*n* = 145).

Variables	Categories	*n* (%) or Mean ± SD
Age(years)	<30	76 (52.4)
	≥30	69 (47.6)
	Mean ± SD	30.61 ± 5.68
Marital status	Unmarried	98 (67.6)
	Married	47 (32.4)
Education level	Associate’s degree	11 (7.6)
	Bachelor’s degree	115 (79.3)
	≥Master’s degree	19 (13.1)
Type of hospital	Tertiary hospital	89 (61.4)
	General hospital	56 (38.6)
Position	Staff nurse	131 (90.3)
	Charge nurse or higher	14 (9.7)
Total clinical experience (years)	<3	44 (30.3)
	3–<5	34 (23.5)
	5–<10	38 (26.2)
	≥10	29 (20.0)
	Mean ± SD	6.45 ± 5.88
NICU experience (years)	<1	21 (14.5)
	1–<3	57 (39.3)
	3–<5	30 (20.7)
	≥5	37 (25.5)
	Mean ± SD	3.74 ± 3.41
Work type	Rotating shift	143 (98.6)
	Fixed shift	2 (1.4)
Number of assigned newborns	≤3	54 (37.2)
	4	66 (45.6)
	≥5	25 (17.2)
Subjective health status	Healthy	53 (36.6)
	Average	76 (52.4)
	Unhealthy	16 (11.0)

**Table 2:** Levels of Resilience, Communication Competence, Nursing Work Environment, and Job Satisfaction (*n* = 145).

Variables	Mean ± SD	Min	Max
Resilience	2.42 ± 0.47	1.32	3.68
Communication Competence	3.82 ± 0.47	2.47	4.93
Nursing Work Environment	2.85 ± 0.41	1.93	3.79
Job Satisfaction	3.58 ± 0.63	2.03	4.91

**Table 3:** Differences in Resilience, Communication Competence, Nursing Work Environment, and Job Satisfaction according to general characteristics (*n* = 145).

Variables	Categories	Resilience			Communication Competence			Nursing Work Environment			Job Satisfaction		
		Mean ± SD	t/F	*p*	Mean ± SD	t/F	*p*	Mean ± SD	t/F	*p*	Mean ± SD	t/F	*p*
Age(years)	<30	2.47 ± 0.47	1.24	0.216	3.91 ± 0.44	2.45	0.016 *	2.93 ± 0.36	2.55	0.012 *	3.60 ± 0.60	0.36	0.721
	≥30	2.37 ± 0.47			3.72 ± 0.48			2.76 ± 0.44			3.56 ± 0.66		
Marital status	Unmarried	2.46 ± 0.46	1.19	0.236	3.88 ± 0.46	2.34	0.021 *	2.89 ± 0.36	1.92	0.057	3.58 ± 0.61	0.12	0.908
	Married	2.36 ± 0.48			3.69 ± 0.48			2.76 ± 0.48			3.57 ± 0.67		
Education level	Associate’s degree	2.52 ± 0.52	1.94	0.148	3.68 ± 0.48	1.10	0.337	2.94 ± 0.28	0.89	0.412	3.62 ± 0.70	1.37	0.256
	Bachelor’s degree	2.39 ± 0.48			3.81 ± 0.45			2.86 ± 0.42			3.54 ± 0.65		
	≥Master’s degree	2.60 ± 0.36			3.94 ± 0.57			2.75 ± 0.37			3.79 ± 0.39		
Type of hospital	Tertiary hospital	2.47 ± 0.41	1.40	0.164	3.83 ± 0.42	0.30	0.769	2.93 ± 0.40	3.30	0.001 **	3.70 ± 0.52	3.17	0.002 **
	General hospital	2.36 ± 0.54			3.80 ± 0.54			2.71 ± 0.40			3.38 ± 0.74		
Position	Staff nurse	2.42 ± 0.47	−0.67	0.507	3.84 ± 0.48	1.57	0.120	2.84 ± 0.42	−0.97	0.337	3.57 ± 0.64	−0.64	0.524
	Charge nurse or higher	2.50 ± 0.49			3.63 ± 0.35			2.95 ± 0.28			3.68 ± 0.50		
Total clinical experience (years)	<3 ^a^	2.54 ± 0.42	2.15	0.097	3.98 ± 0.40	2.53	0.060	3.00 ± 0.32	3.74	0.013 *	3.66 ± 0.51	2.18	0.094
	3–<5 ^b^	2.32 ± 0.50			3.73 ± 0.50			2.78 ± 0.42		(d < a)	3.38 ± 0.68		
	5–<10 ^c^	2.33 ± 0.52			3.78 ± 0.50			2.73 ± 0.45			3.54 ± 0.75		
	≥10 ^d^	2.48 ± 0.38			3.74 ± 0.46			2.85 ± 0.42			3.74 ± 0.50		
NICU experience (years)	<1	2.53 ± 0.37	0.90	0.446	3.91 ± 0.36	2.21	0.089	3.04 ± 0.45	2.31	0.079	3.56 ± 0.61	1.13	0.339
	1–<3	2.42 ± 0.50			3.91 ± 0.45			2.86 ± 0.45			3.52 ± 0.68		
	3–<5	2.32 ± 0.45			3.67 ± 0.51			2.79 ± 0.32			3.50 ± 0.58		
	≥5	2.46 ± 0.48			3.75 ± 0.50			2.77 ± 0.37			3.74 ± 0.59		
Work type	Rotating shift	2.42 ± 0.47	−0.41	0.680	3.82 ± 0.47	0.56	0.575	2.85 ± 0.41	1.34	0.183	3.58 ± 0.63	−0.03	0.976
	Fixed shift	2.56 ± 0.23			3.63 ± 0.24			2.47 ± 0.27			3.59 ± 0.24		
Number of assigned newborns	≤3	2.42 ± 0.47	0.94	0.392	3.81 ± 0.39	0.21	0.808	2.89 ± 0.41	2.45	0.090	3.58 ± 0.59	0.41	0.663
	4	2.46 ± 0.48			3.85 ± 0.56			2.87 ± 0.44			3.61 ± 0.69		
	≥5	2.31 ± 0.45			3.78 ± 0.38			2.69 ± 0.30			3.48 ± 0.54		
Subjective health status	Healthy ^a^	2.62 ± 0.40	13.82	<0.001 ***	3.94 ± 0.41	6.83	0.001 **	2.89 ± 0.36	5.49	0.005 **	3.74 ± 0.55	7.96	0.001 **
	Average ^b^	2.38 ± 0.45		(c < b < a)	3.81 ± 0.46		(c < a,b)	2.88 ± 0.41			3.57 ± 0.61		(c < a,b)
	Unhealthy ^c^	1.99 ± 0.46			3.46 ± 0.53			2.54 ± 0.45			3.06 ± 0.71		

Note. Post hoc comparisons were conducted using Scheffé’s test. Different letter notations indicate significant differences among groups; * *p* < 0.05, ** *p* < 0.01, *** *p* < 0.001.

**Table 4:** Correlations among Resilience, Communication Competence, Nursing Work Environment, and Job Satisfaction (*n* = 145).

Variables	1	2	3	4
1. Resilience	1			
2. Communication Competence	0.61 ***	1		
3. Nursing Work Environment	0.44 ***	0.35 ***	1	
4. Job Satisfaction	0.67 ***	0.52 ***	0.57 ***	1

*** *p* < 0.001.

**Table 5:** Factors associated with Job Satisfaction (*n* = 145).

Variables	B	SE	*β*	t	*p*
Resilience	0.52	0.08	0.43	6.45	<0.001
Nursing work environment	0.47	0.09	0.30	5.02	<0.001
Communication competence	0.21	0.10	0.15	2.07	0.040
Tertiary hospital (ref: general)	0.18	0.09	0.12	2.09	0.038
Subjective health status (ref: unhealthy)					
Healthy	0.03	0.13	0.03	0.26	0.799
Average	0.03	0.12	0.02	0.24	0.814

R^2^ = 0.569, Adjusted R^2^ = 0.551, F = 30.42, *p* < 0.001.

## Data Availability

The data presented in this study are not publicly available due to privacy and ethical restrictions but are available from the corresponding author upon reasonable request.

## Abbreviations

The following abbreviations are used in this manuscript:

GICC	Global Interpersonal Communication Competence Scale
K-CD-RISC	Korean version of the Connor–Davidson Resilience Scale
NICU	Neonatal Intensive Care Unit
PES-NWI	Practice Environment Scale of the Nursing Work Index
JSS-CN	Job Satisfaction Scale for Clinical Nurses
